# Bioinformatic survey of CRISPR loci across 15 *Serratia* species

**DOI:** 10.1002/mbo3.1339

**Published:** 2023-03-02

**Authors:** Maria Scrascia, Roberta Roberto, Pietro D'Addabbo, Yosra Ahmed, Francesco Porcelli, Marta Oliva, Carla Calia, Angelo Marzella, Carlo Pazzani

**Affiliations:** ^1^ Department of Biology University of Bari Aldo Moro Bari Italy; ^2^ Dipartimento di Scienze del Suolo, della Pianta e degli Alimenti University of Bari Aldo Moro Bari Italy; ^3^ Plant Quarantine Pathogens Laboratory, Mycology Research & Disease Survey Plant Pathology Research Institute, ARC Giza Egypt

**Keywords:** CRISPR system, *Rhynchophorus ferrugineus*, RPW, subtype I‐C, subtype I‐E, subtype I‐F1

## Abstract

The Clustered Regularly Interspaced Short Palindromic Repeats and CRISPR‐associated proteins (CRISPR–Cas) system of prokaryotes is an adaptative immune defense mechanism to protect themselves from invading genetic elements (e.g., phages and plasmids). Studies that describe the genetic organization of these prokaryotic systems have mainly reported on the Enterobacteriaceae family (now reorganized within the order of Enterobacterales). For some genera, data on CRISPR–Cas systems remain poor, as in the case of *Serratia* (now part of the *Yersiniaceae* family) where data are limited to a few genomes of the species *marcescens*. This study describes the detection, in silico, of CRISPR loci in 146 *Serratia* complete genomes and 336 high‐quality assemblies available for the species *ficaria*, *fonticola*, *grimesii*, *inhibens*, *liquefaciens*, *marcescens*, *nematodiphila*, *odorifera*, *oryzae*, *plymuthica*, *proteomaculans*, *quinivorans*, *rubidaea*, *symbiotica*, and *ureilytica*. Apart from subtypes I‐E and I‐F1 which had previously been identified in *marcescens*, we report that of I‐C and the I‐E unique locus 1, I‐E*, and I‐F1 unique locus 1. Analysis of the genomic contexts for CRISPR loci revealed *mdtN*‐*phnP* as the region mostly shared (*grimesii*, *inhibens*, *marcescens*, *nematodiphila*, *plymuthica*, *rubidaea*, and *Serratia* sp.). Three new contexts detected in genomes of *rubidaea* and *fonticola* (*puu* genes‐*mnmA*) and *rubidaea* (*osmE*‐*soxG* and *ampC*‐*yebZ*) were also found. The plasmid and/or phage origin of spacers was also established.

## Graphical abstract

This study has contributed to extending the general knowledge of the CRISPR–Cas systems, particularly in Serratia, a ubiquitous genus detected in different environments.

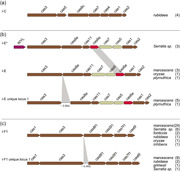

## 1 INTRODUCTION

The prokaryotic system Clustered Regularly Interspaced Short Palindromic Repeats and CRISPR‐associated proteins (CRISPR–Cas) is a defense mechanism for bacteria and archaea against the invasion of bacteriophages and selfish genetic elements such as plasmids. Since their discovery around 15 years ago (Bolotin et al., 2005; Makarova et al., 2006; Mojica et al., 2005), CRISPR–Cas systems have been the object of many studies and functions, other than adaptative immunity, as regulation of bacteria virulence and stress response have been reported (Faure et al., 2019; Louwen et al., 2014). Based on a census of complete genomes, it is now reckoned that these systems are distributed mainly in archaea (~82.5%) and, to a lesser extent, bacteria (~40%) (Makarova et al., 2020). The CRISPR‐–Cas systems are composed of CRISPR arrays and adjacent CRISPR‐associated (cas) genes. The former are composed of direct repeats interspaced by spacers; the latter encode proteins involved in the immune response and DNA repair. This ever‐expanding knowledge of the composition and architecture of cas gene clusters has led to an updated classification of CRISPR–Cas systems where two classes, six types, and various subtypes (some of which are further divided into different variants) are now reported (Koonin & Makarova, 2017; Makarova et al., 2020). Class 1 includes the types I (DNA targeting), III (DNA and/or RNA targeting), and IV (DNA targeting), which are divided into seven subtypes I (A–G), six subtypes III (A–F), and three subtypes IV (A–C), respectively. Class 2 includes the types II (DNA targeting), V (DNA or RNA targeting), and VI (RNA targeting); they are also divided into subtypes: three subtypes II (A–C), eleven subtypes V (A–K and U), and four subtypes VI (A–D), respectively (Koonin & Makarova, 2017; Makarova et al., 2020). While Class 2 is found mainly in Bacteria, Class 1 is present both in Bacteria and Archaea. Studies on CRISPR–Cas systems have been performed on genomes of different bacteria families, with that of the *Enterobacteriaceae* being one of the most investigated (Medina‐Aparicio et al., 2018; Shariat & Dudley, 2014; Xue & Sashital, 2019). This family was unique in the *Enterobacterales* order until 2016 when Adeolu et al. (2016) reclassified the order by adding six new families (*Budviciaceae*, *Erwiniaceae*, *Hafniaceae*, *Morganellaceae*, *Pectobacteriaceae*, *Yersiniaceae*). Despite this reclassification, data on CRISPR–Cas systems remain mainly limited to genera of the *Enterobacteriaceae* family (Díez‐Villaseñor et al., 2010; Shariat et al., 2015; Shen et al., 2017; Wang et al., 2016).

The genus *Serratia*, a Gram‐negative rod, is now part of the family *Yersiniaceae*. *Serratia* species can be found in different environments (e.g., water, soil) and hosts (e.g., humans, insects, plants, vertebrates) where they may play different roles ranging from opportunistic pathogens to symbionts (Cristina et al., 2019; Gupta et al., 2021; Lo et al., 2016). Among *Serratia* species, *marcescens* is undoubtedly the most studied mainly for its role played as a symbiont associated with insects and nematodes (Chen et al., 2017) or as a human opportunistic pathogen (currently reported as one of the most important bacteria responsible for acquired hospital infections such as bacteremia, pneumonia, intravenous catheter‐associated infections, and endocarditis) (Ferreira et al., 2020). Other *Serratia* species responsible (to *a minor* extent) for human bacteremia are *liquefaciens* and *odorifera* (Mahlen, 2011). A growing number of *marcescens* genomes have then been sequenced with a pangenome allele database available for different studies ranging from virulence and antibiotic resistance to the identification of CRISPR systems (Abreo & Altier, 2019). A number of studies, in addition to *marcescens*, have also been reported for other *Serratia* species that play different roles in human and insect pathogenesis(Petersen & Tisa, 2013). Although the characterization of CRISPR systems represents a valuable substrate for diagnostic, epidemiologic, and evolutionary analyses (Louwen et al., 2014), data on CRISPR–Cas systems in the genus are scarce and limited to the detection of subtypes I‐E and I‐F1 in genomes of the species *marcescens* (Medina‐Aparicio et al., 2018; Scrascia et al., 2019; Srinivasan & Rajamohan, 2019; Vicente et al., 2016).

In this study, 146 *Serratia* complete genomes and 336 high‐quality assemblies are available for the species *ficaria*, *fonticola*, *grimesii*, *inhibens*, *liquefaciens*, *marcescens*, *nematodiphila*, *odorifera*, *oryzae*, *plymuthica*, *proteomaculans*, *quinivorans*, *rubidaea*, *symbiotica*, and *ureilytica* were explored for the presence and type of cas gene clusters and/or CRISPRs. Apart from subtypes I‐E and I‐F1, the study showed the presence (first detected in *Serratia*) of subtype I‐C, the presence of unique loci, and detailed genomic contexts of CRISPR loci. The plasmid and/or phage origin of spacers was also assessed.

The discovery of CRISPR–Cas systems has allowed the development of new technology tools in the bioengineering field (Dong et al., 2021). A clear example is represented by gene editing strategies based on CRISPR/Cas9 technique successfully used in agriculture, nutrition, and human health (Nidhi et al., 2021). The development of new CRISPR‐based applications also relies on the continuous update of CRISPR–Cas systems data and knowledge. Our study, in providing more comprehensive data on CRISPR loci in *Serratia*, has undoubtedly contributed to an expanded knowledge of these systems.

## 2 MATERIALS AND METHODS

### 2.1 Genomes analyzed

One hundred and forty‐six *Serratia* complete genomes were considered in this study. The set of genomes encompasses the 15 *S. marcescens* complete genomes we previously analyzed (Scrascia et al., 2019) and those of the genus *Serratia* available at the CRISPR–Cas^++^ database (https://crisprcas.i2bc.paris-saclay.fr/MainDb/StrainList) up to December 12, 2020 (Couvin et al., 2018; Pourcel et al., 2020) (Supporting Information: Table S1). Among genome sequences available at the assembly level of scaffolds or contigs available at the National Center for Biotechnology Information database (NCBI) (https://www.ncbi.nlm.nih.gov/assembly) up to December 12, 2020, we selected the high‐quality assemblies (N50 > 50 kb, i.e. 50% of the entire assembly is contained in contigs or scaffolds equal to or larger than the 50 kb) that have been included in the study.

Species attribution and strain details (name, place, date of isolation) were recovered (when available) from GenBank or related articles. *Serratia* strains AS12 (NC_015566.1), FGI94 (NC_020064), FS14 (NZ_CP005927), SCBI (NZ_CP003424), YD25 (NZ_CP016948), and DSM21420 (GCA_000738675) were reclassified as reported by Sandner‐Miranda et al. (2018), Sandner‐Miranda et al. (2018). In the study reported by Sandner‐Miranda et al., the strain ATCC39006 was not assigned to the genus *Serratia* and we did not include it in this study.

We also included sequences with the accessions MK507743, MK507744, MK507745, and MK507746 referring to contigs (N50 ranging from 228817 to 291462) harboring CRISPR loci in genome assemblies (unpublished) of four *S. marcescens* strains reported as secondary symbionts in the Red Palm Weevil (RPW) *Rhynchophorus ferrugineus* (Olivier, 1790) (Coleoptera: Curculionidae) (Scrascia et al., 2016, 2019) (Supporting Information: Table S1), an alien invasive pest now threatening South America (Dalbon et al., 2021).

### 2.2 Detection of CRISPR–Cas *loci*

Details about the detection of a cas gene cluster with associated arrays (CRISPR–Cas system) and CRISPR arrays only for complete genomes were retrieved from the CRISPR–Cas^*++*^ database. CRISPR arrays recorded by CRISPR–Cas^++^ were assigned to Levels 1–4 based on the criteria required to select the minimal structure of putative CRISPR as reported by Pourcel et al. (2020). Level 1 is the lowest level of confidence. Levels 2–4 were assigned based on the conservation of repeats (which must be high in a real CRISPR) and on the similarity of spacers (it must be low). Level 4 CRISPRs were defined as the most reliable ones. Levels 1–3 may correspond to false CRISPRs. In our study, only CRISPRs recorded with Level 4, were considered. CRISPRs without a set of cas genes in the host genome were defined as “orphans.” Genomes harboring cas gene clusters were then submitted to the CRISPRone analysis suite (http://omics.informatics.indiana.edu/CRISPRone/) (Zhang & Ye, 2017) to graphically visualize the architecture of each cluster. The same suite was used to search and visualize cas gene clusters in the high‐quality assemblies. A subtype of cas gene clusters was assigned according to the recent classification update for CRISPR–Cas systems (Makarova et al., 2020).

### 2.3 In silico analyses of consensus of direct repeats

**Figure 1:**
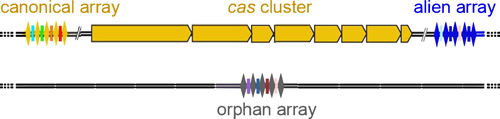
Schematic diagram of the three categories of arrays described in the study. DRs and spacers are depicted with diamonds and rectangles respectively. *cas* genes are shown as arrows pointing in the direction of transcription. The yellow color highlights the consistency between the DR type and the cas subtype; while the blue color indicates inconsistency.

A consensus of direct repeats from CRISPRs was clustered by BLAST similarity. Some consensus DRs were manually trimmed when just a few terminal nucleotides were the only difference from the other members of the same cluster. The consensus DRs were used as input for CRISPRBank (http://crispr.otago.ac.nz/CRISPRBank/index.html) and CRISPR–Cas^++^ to assign, based on identity with known consensus DRs (Biswas et al., 2016; Couvin et al., 2018; Pourcel et al., 2020), a specific CDR type to CRISPR. The CRISPRs whose CDR type was consistent with the subtype of the cas gene set harbored in the same genome were defined as “canonical.” While those not consistent with the subtype of the cas gene set harbored in the same genome were defined as “alien.” A schematic diagram of alien, canonical and orphan arrays is shown in Figure 1. consensus DRs and the number of repeats of the CRISPRs in the high‐quality assemblies of *Serratia* sp. strains DD3, Ag1, and Ag2 were recovered from the CRISPRone output. Spacers’ analysis for duplications (spacers of Ag1, Ag2, and DD3 included) was performed through the CRISPRCasdb spacer database at the CRISPRCas^++^ site (https://crisprcas.i2bc.paris-saclay.fr/MainDbQry/Index). Phagic and/or plasmidic origin of matching protospacers were searched at the CRISPRTarget site (http://crispr.otago.ac.nz/CRISPRTarget/crispr_analysis.html) (Biswas et al., 2016).

### 2.4 Genomic contexts of CRISPR‐positive genomes

Analysis of CRISPR‐positive complete genomes and high‐quality assemblies was performed to better characterize the genomic context surrounding the cas gene sets and/or CRISPR arrays. High‐quality assemblies with at least 4 kb flanking the cas gene sets were considered. These regions were annotated by Prokka (https://github.com/tseemann/prokka) (Seemann, 2014). Synteny was established by either the Mauve algorithm (http://darlinglab.org/mauve/mauve.html) (Darling et al., 2010) or visual inspection of annotated proteins.

### 2.5 Phylogenetic analyses

The evolutionary relationship of *Serratia* strains found positive for cas genes sets was established and graphically depicted by the Cas3 sequence tree. All the protein sequences were aligned by the MUSCLE algorithm (https://www.ebi.ac.uk/Tools/msa/muscle/) (Edgar, 2004a, 2004b). The 16S rRNA gene tree was also drawn for comparison. Dendrograms were generated by the Neighbor‐Joining clustering method and average distance trees with JalView (https://www.jalview.org/) (Waterhouse et al., 2009). For the 16S rRNA gene tree, the multiple sequence alignment was obtained by retrieving from one to seven full gene sequences (complete genomes) or truncated 16S rRNA gene sequences (high‐quality assemblies). A phylogenetic tree was obtained by multiple alignment of all retrieved 16S rRNA genes; an abbreviated tree was constructed by using one sequence from each genome.

## 3 RESULTS

### 3.1 CRISPR‐positive genomes

**Figure 2:**
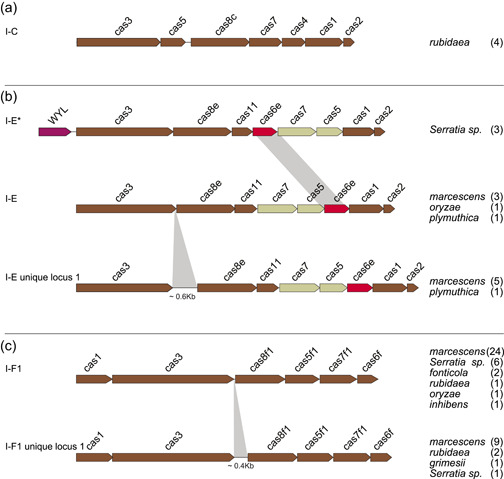
Architectures of canonical and unique cas gene sets. Genes are shown as arrows pointing in the direction of transcription. Gray shadows highlight the distinguishing features of the I‐E unique locus 1, I‐E*, and I‐F1 unique locus 1. Species in which the architectures were detected are reported on the right side and the number of genomes is reported in brackets. Truncated *cas* gene sets (due to the end of contigs) were not shown. (a) Genetic organization of the canonical *cas* gene set I‐C. (b) Genetic organization of *cas* gene sets for the canonical I‐E, the I‐E*, and I‐E unique locus 1. The WYL domain is highlighted as a red arrow. (c) Genetic organization of *cas* gene sets for the canonical I‐F1 and the I‐F1 unique locus 1.

A collection of 146 *Serratia* complete genomes was explored for the presence of cas gene clusters and/or CRISPR arrays. Most of the genomes (134) were reported as known species: *ficaria* (1), *fonticola* (7), *grimesii* (1), *inhibens* (1), *liquefaciens* (7), *marcescens* (87), *nematodiphila* (1), *plymuthica* (11), *proteomaculans* (2), *quinivorans* (2), *rubidaea* (8), *symbiotica* (4), *ureilytica* (2). The remaining 12 genomes were of unidentified species and, from here on, they will be referred to as *Serratia* sp. (Supporting Information: Table S1). **The** CRISPR–Cas systems or only CRISPR arrays (orphan array) were detected in 35 complete genomes (24%) of which 17 harbored a CRISPR–Cas system, while 18 harbored orphan arrays. Some complete genomes characterized by the same cas gene set subtype and identical numbers of both CRISPRs and spacers were assumed as multiple records of the same genome (Table 1). All detected cas gene clusters were of Class 1. Nine were identical to those already published (Makarova et al., 2020) and distributed as follows: two subtypes I‐C (*rubidaea*) (Figure 2a), one I‐E (*plymuthica*) and six I‐F1 (1 *fonticola*, 3 *marcescens*, 1 *inhibens*, and 1 *rubidaea*) (Figure 2b,c). The remaining eight clusters were found atypical and assigned, in this study, to I‐E unique locus 1 (3 *marcescens* and 1 *plymuthica*) and I‐F1 unique locus 1 (1 *marcescens*, 2 *rubidaea*, and 1 *Serratia* sp.).

**Table 1:** *Cas* genes clusters and CRISPRs in complete genomes

Subtype of cas cluster	CRISPRs			*Serratia* species	Strain	Source	Place of isolation	Year of isolation	Accession/Assembly
	CDR type	Category	#Arrays (#spacers)						
I‐C	I‐C	Canonical	1 (14)	*rubidaea*	FDAARGOS_926^a^	N/A	N/A	N/A	NZ_CP065640.1
	I‐E	Alien	1 (7)						
	I‐F	Alien	2 (2, 5)						
I‐C	I‐C	Canonical	1 (14)	*rubidaea*	NCTC12971^a^	N/A	N/A	N/A	LR590463.1
	I‐E	Alien	1 (7)						
	I‐F	Alien	2 (2, 5)						
I‐E	I‐E	Canonical	2 (43, 30)	*plymuthica*	NCTC8900	N/A	N/A	N/A	LR134151.1
I‐E unique locus 1	I‐E	Canonical	4 (6, 8, 27, 44)	*marcescens*	E28	Hospital Ensuite	Australia	2012	CP042512.1
“	I‐E	Canonical	3 (7, 10, 22)	*marcescens*	SER00094	Clinical	United States	2017	CP050447.1
“	I‐E	Canonical	3 (11, 39, 69)	*marcescens*	MSB1_9C‐sc‐2280320	N/A	N/A	N/A	LR890657.1
“	I‐E	Canonical	2 (35, 47)	*plymuthica*	NCTC8015	Canal water	N/A	N/A	LR134478.1
I‐F1	I‐F	Canonical	2 (25, 27)	*marcescens*	12TM	Pharyngeal secretions	Romania	2014	CM008894.1
I‐F1	I‐F	Canonical	2 (8, 17)	*marcescens*	N4‐5	Soil	United States	1995	CP031316.1
I‐F1	I‐F	Canonical	2 (6, 45)	*marcescens*	PWN146	*Bursaphelenchus xylophilus*	Portugal	2010	LT575490.1
I‐F1	I‐F	Canonical	3 (11, 13, 42)	*fonticola*	DSM 4576	Water	N/A	1979	NZ_CP011254.1
I‐F1	I‐F	Canonical	2 (15, 24)	*inhibens*	PRI‐2c	Maize rhizosphere soil	The Netherlands	2004	NZ_CP015613.1
I‐F1	I‐F	Canonical	6 (1, 3, 7, 7, 14, 14)	*rubidaea*	FDAARGOS_880	N/A	N/A	N/A	CP065717.1
I‐F1 unique locus 1	I‐F	Canonical	3 (5, 10, 29)	*marcescens*	FZSF02	soil	China	2014	CP053286
“	I‐E	Alien	1 (9)	*rubidaea*	FGI94	*Atta colombica*	Panama	2009	NC_020064.1;
	I‐F	Canonical	3 (6, 15, 16)						CP003942
“	I‐F	Canonical	4 (3, 6, 7, 8)	*rubidaea*	NCTC10036	Finger	N/A	N/A	LR134493.1
	I‐E	Alien	1 (3)						
“	I‐F	Canonical	4 (2, 2, 7, 7, 10)	*Serratia* sp.	JUb9	Compost	France	2019	CP060416.1
N/A	I‐F	Orphan	1 (21)	*marcescens*	SCQ1	Blood from silkworm	China	2009	CP063354.1
N/A	I‐F	Orphan	1 (3)	*marcescens*	AR_0130	N/A	N/A	N/A	CP028947.1
N/A	I‐F	Orphan	1 (6)	*plymuthica*	AS9^b^	Plant	Sweden	N/A	NC_015567.1; CP002773.1
N/A	I‐F	Orphan	1 (6)	*plymuthica*	AS12^b^	Plant	Sweden	1998	NC_015566.1; CP002774
N/A	I‐F	Orphan	1 (6)	*plymuthica*	AS13^b^	Plant	Sweden	N/A	NC_017573.1; CP002775
N/A	I‐F	Orphan	1 (3)	*marcescens*	B3R3	*Zea mays*	China	2011	NZ_CP013046.2
N/A	I‐F	Orphan	2 (1, 2)	*Serratia* sp.	MYb239	Compost	Germany	N/A	CP023268.1
N/A	I‐F	Orphan	1 (3)	*Serratia* sp.	SSNIH1	N/A	United States	2015	CP026383.1
N/A	I‐F	Orphan	1 (3)	*nematodiphila*	DH‐S01	N/A	N/A	N/A	CP038662.1
N/A	I‐F	Orphan	2 (4, 6)	*rubidaea*	NCTC9419	N/A	N/A	N/A	LR134155.1
N/A	I‐F	Orphan	2 (6, 2)	*rubidaea*	NCTC10848	N/A	N/A	N/A	LS483492.1
N/A	I‐E	Orphan	1 (3)						
N/A	I‐E	Orphan	1 (26)	*marcescens*	KS10^c^	Marine	United States	2006	CP027798.1
N/A	I‐E	Orphan	1 (26)	*marcescens*	EL1^c^	Marine	United States	2002	CP027796.1
N/A	I‐E	Orphan	2 (3, 32)	*marcescens*	CAV1761^d^	Peri‐rectal	Virginia	2014	CP029449.1
N/A	I‐E	Orphan	2 (3, 32)	*marcescens*	CAV1492^d^	Clinical	United States	2011–2012	NZ_CP011642.1
N/A	I‐E	Orphan	1 (2)	*Serratia* sp.	KUDC3025	Rhizospheric soil	South Korea	2017	CP041764.1
N/A	I‐F	Orphan	1 (2)	*plymuthica*	V4	Milk processing plant	Portugal	2006	CP007439.1
N/A	I‐C	Orphan	1 (8)	*symbiotica*	CWBI‐2.3	*Aphis fabae* (type strain of S. symbiotica)	Belgium	2009	CP050855.1

Abbreviations: CDR, consensus DR; CRISPR– Cas, Clustered Regularly Interspaced Short Palindromic Repeats and CRISPR‐associated proteins.

^a,b,c,d^ Possible multiple records of the same genome.

**Figure 3:**
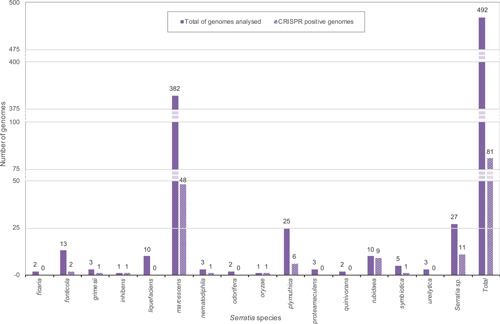
Distribution of CRISPR‐positive genomes. Solid boxes represent the total number (top of boxes) of genomes analyzed per species. Dashed boxes show the number (top of boxes) of genomes for which CRISPR–Cas systems or CRISPRs were detected.

The I‐E unique locus 1 had the *cas3‐cas8e* genes spaced by ~600 nt while the I‐F1 unique locus 1 had the *cas3*‐*cas8f1* genes separated from each other by ~400 nt (Figure 2b,c). Since the I‐E unique locus 1 and the I‐F1 unique locus 1 cas gene clusters have never been reported in *Serratia*, their presence was further explored among 336 *Serratia* high‐quality assemblies. The assemblies were distributed as follows: *ficaria* (1), *fonticola* (6), *grimesii* (2), *liquefaciens* (3), *marcescens* (295), *nematodiphila* (2), *odorifera* (2), *oryzae* (1), *plymuthica* (4), *proteomaculas* (1), *rubidaea* (2), *symbiotica* (1), *ureilytica* (1), and *Serratia* sp. (15) (Supporting Information: Table S1). Of the 336 analyzed genomes, 46 (13.7%) were positive for the presence of cas gene clusters. Twenty‐six were subtype I‐F1 (21 *marcescens*, one *fonticola*, and 4 *Serratia* sp.) (Figure 2c), two subtype I‐C (*rubidaea*) (Figure 2a), and three subtype I‐E (*marcescens*) (Figure 2b; Table A1). The I‐E unique locus 1 was detected in two genomes of *marcescens*, the I‐F1 unique locus 1 in eight genomes of *marcescens*, and one of *grimesii*. In three genomes of *Serratia* sp. (strains Ag1, Ag2, and DD3) an additional unique locus of the subtype I‐E, identical to I‐E* previously reported by Shen et al. (2017), was detected (Figure 2b). The locus I‐E* identified in this study was characterized by the translocation of *cas6e* between *cas7* and *cas11*, and the presence (upstream of *cas3*) of a gene harboring the WYL domain which encodes for a potential functional partner of the CARF (CRISPR–Cas Associated Rossmann Fold) superfamily proteins (Makarova et al., 2020). Proteins containing the WYL domain (name standing for the three conserved amino acids tryptophan, tyrosine, and leucine, respectively) have only been reported for subtypes I‐D and VI‐D (Makarova et al., 2014, 2019). The distribution of CRISPR‐positive genomes, over the total analyzed, among *Serratia* species is shown in Figure 3. Coexistence in the same genome of different sets of cas genes was also detected: subtypes I‐E and I‐F1 were found in the single HQA of *oryzae*, while I‐E* and I‐F1were detected in two high‐quality assemblies of *Serratia* sp. (strains Ag1 and Ag2) (Table A1).

### 3.2 Consensus DRs and spacers

The 35 CRISPR‐positive complete genomes harbored 78 CRISPRs of which 48 were canonical. The latter were distributed as follows: *fonticola* (4), *inhibens* (1), *marcescens* (19), *plymuthica* (5), *rubidaea* (15), and *Serratia* sp. (4). Twenty‐three arrays were orphans and detected in genomes of *marcescens* (8), *plymuthica* (4), *symbiotica* (1), *nematodiphila* (1), *rubidaea* (5), and *Serratia* sp. (4) (Table 1; Figure 1). Alien arrays (8) were only detected in the species *rubidaea*. For a comprehensive analysis, arrays in the three high‐quality assemblies Ag1, Ag2, and DD3 were included (Table A1). All disclosed CRISPRs were assigned, by comparative sequence analyses, to consensus DR types I‐C, I‐E, or I‐F (Table 1). The association between consensus DR types and cas gene sets (canonical and unique loci) is reported in Table 2. Based on their nucleotide identity, the consensus DRs identified for subtype I‐E and its unique loci (I‐E* and unique locus 1) could be arranged into two clusters named consensus DR‐I and consensus DR‐II. consensus DR‐I was composed of 6 consensus DRs (identity from 83% to 96%) and linked to the cas gene sets I‐E and I‐E unique locus 1. consensus DR‐II was composed of 2 consensus DRs (identity of about 96%) and linked to the cas gene set I‐E*. When the consensus DRs of the two clusters were compared to each other, the nucleotide identity dropped to 55%–62%.

**Table 2:** Association between consensus DRs and *cas* gene sets

Sequence (5'−3')	# nt	Record in CRISPRBank and CRISPR–Cas^++^	CDR^a^ type	Associated cas genes set(s)
GTCGTGCCTCATGCAGGCACGTGGATTGAAAC	32	I‐C	I‐C	I‐C
GTCGTGCCTCACGTAGGCACGTGGATTGAAA	31	I‐C	I‐C	I‐C
CGGTTCATCCCCGCTGGCGCGGGGAATAG^a^^,^ d	29	I‐E	I‐E	I‐E
CGGTTTATCCCCGCTCTCGCGGGGAACAC^a^	29	I‐E	I‐E	I‐E; I‐E unique locus 1
CGGTTTATCCCCGCTGACGCGGGGAACAC^a^	29	I‐E	I‐E	I‐E unique locus 1
CGGTTTATCCCCGCTGGCGCGGGGAACAC^a^	29	I‐E	I‐E	I‐E; I‐E unique locus 1
CGGTTTATCCCCGCTCGCGCGGGGAACAC^a^	29	I‐E	I‐E	I‐E
CGGTTTATCCCCGCTAGCGCGGGGAACAC^a^	29	I‐E	I‐E	I‐E
GAAACACCCCCACGTGCGTGGGGAAGAC^b^^,^ c	28	I‐E	I‐E*	I‐E*
GAAACACCCCCACGTGCGTGGGGAAGGC^d^^,^ c	28	I‐E	I‐E*	I‐E*
GTGCACTGCCGTACAGGCAGCTTAGAAA	28	I‐F	I‐F	I‐F1; I‐F1 unique locus 1
GTTCACTGCCGCATAGGCAGCTTAGAAA	28	I‐F	I‐F	I‐F1
GTTCACTGCCGTGCAGGCAGCTTAGAAA	28	I‐F	I‐F	I‐F1
GTTCACTGCCGTATAGGCAGCTTAGAAA	28	I‐F	I‐F	I‐F1
GTTCGCTGCCGTGCAGGCAGCTTAGAAA	28	I‐F	I‐F	I‐F1
GTTCACTGCCGTACAGGCAGCTTAGAAA	28	I‐F	I‐F	I‐F1

*Note*: Palindrome identified in each consensus DR is underlined.

Abbreviation: CDR, consensus DR.

^a^ Consensus DR‐I group.

^b^ Consensus DR associated with the 20DRs array in Ag1 strain, the 3DRs array in Ag2 strain and the DD3 arrays (Table A1).

^c^ Consensus DR‐II group.

^d^ Consensus DR associated with the 5DRs arrays in Ag1 and Ag2 strains (Table A1).

The architecture of the cas gene set I‐E* has previously been reported for *Klebsiella* and *Vibrio cholerae* (I‐E variant) (McDonald et al., 2019; Shen et al., 2017). We then compared the consensus DRs sequences I‐E* and I‐E variant with those of consensus DR‐II and the identity was found between 82% and 96%. This association has further been confirmed by results obtained from the analysis of the cas gene clusters identified in 99 genomes retrieved from CRISPRBank and by searching for the presence of consensus DRs I‐E*. Results showed that 95 of these genomes had a cas gene architecture identical to that of I‐E*. The remaining four genomes harbored a truncated set of cas genes. Overall these data linked specifically consensus DR‐II to the cas gene set I‐E*.

A total of 1391 spacers were identified. Identical arrays were shared by *rubidaea* strains FDAARGOS_926 and NCTC12971. Likewise, different sets of identical arrays were shared by *plymuthica* strains AS9, AS12, and AS13; *marcescens* strains KS10 and EL1; *marcescens* strains CAV1761 and CAV1492 (Supporting Information: Table S2). These findings confirmed multiple records of the same genome for each group of strains and the total number of spacers was estimated at 1290 of which 1219 were unique and 330 matched protospacers with the following origin: 131 phages, 132 plasmids, and 67 phage/plasmid (Supporting Information: Table S2).

### 3.3 Phylogenetic trees

**Figure 4:**
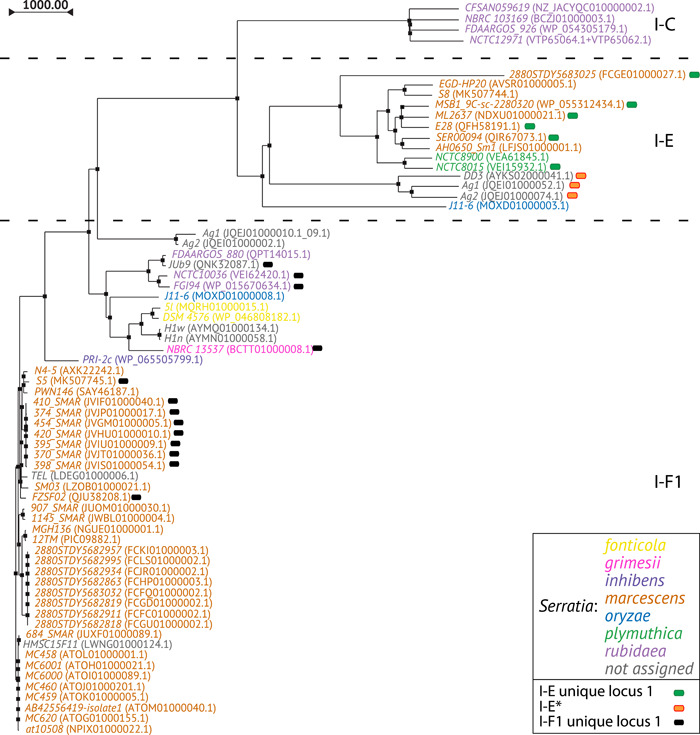
Cas3 phylogenetic tree. Species are shown with different colors. In brackets, the accession number of the *cas3* nucleotide sequence is reported.

**Figure 5:**
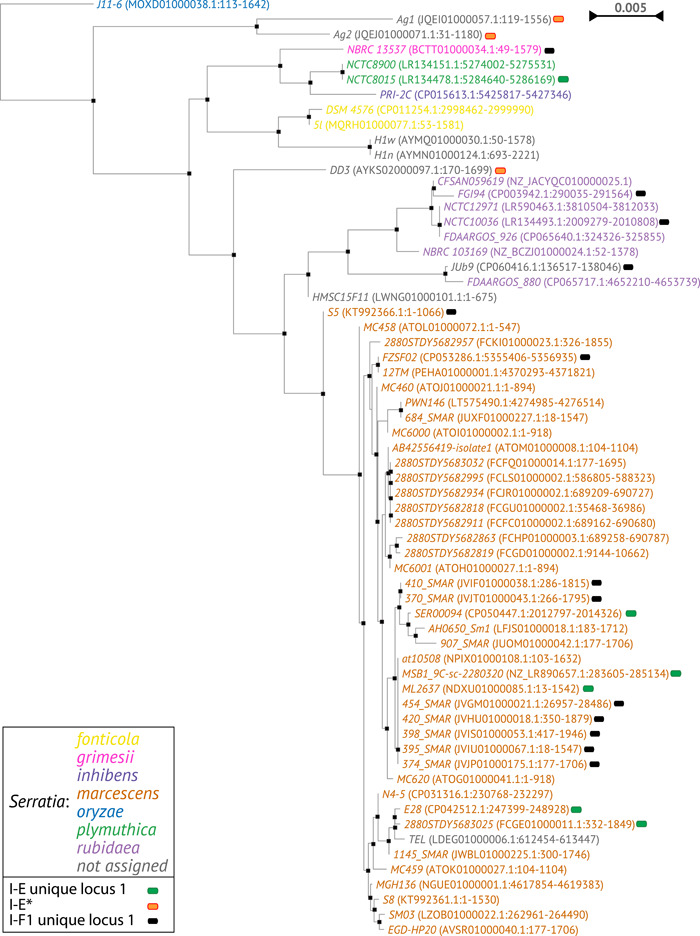
16S rRNA gene phylogenetic tree. Species are shown with different colors. In brackets, the accession number of the 16S rRNA gene nucleotide sequence is reported.

The phylogenetic tree generated by multiple alignment of the amino acid sequences of Cas3 showed a clusterization of the subtypes I‐C, I‐E, and I‐F1 into three distinct branches (Figure 4). The I‐E unique locus 1 and I‐F1 unique locus 1 were randomly distributed among the I‐E and I‐F1, respectively, while the I‐E* appears in a group within a sub‐lineage of I‐E. Within the I‐C, I‐E, and I‐F1 branches, strains from the same species are grouped together. The phylogenetic tree based on multiple alignment of the 16S rRNA gene sequences was generated for comparison (Figure 5 and Supporting Information: Figure S1). The 16S rRNA gene trees showed, as expected, a nesting of the strains from the same species. The phylogenetic distribution of *Serratia* species in the Cas3 tree may suggest a possible independent intra‐species evolutionary pathway. However, because the number of available CRISPR‐positive genomes is too low for most *Serratia* species such a hypothesis needs to be validated by future studies. The position of strains TEL in the cluster *marcescens* and JUb9 in the cluster *rubidaea* shown in the Cas3 phylogenetic tree was confirmed by the 16S rRNA gene tree, which might suggest a species assignment for these strains.

### 3.4 CRISPR genomic contexts

**Figure 6:**
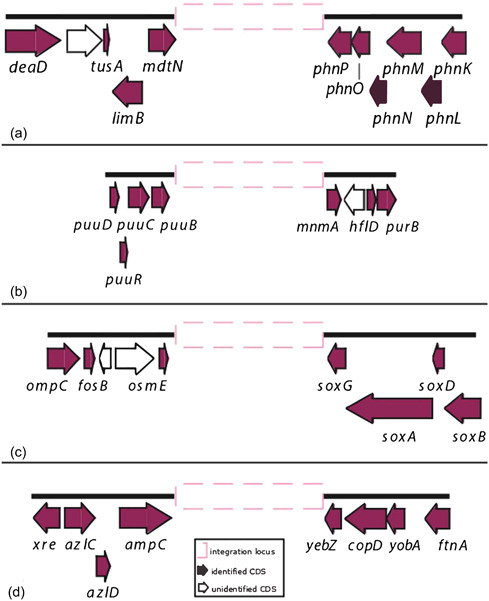
Schematic diagram of the shared genomic contexts A to D. Letters on the left (a–d) indicate the type of genomic context. The pink dashed box represents the genomic region harboring cas set and/or CRISPR arrays. Black thick lines depict flanking regions. Genes are shown as arrow boxes pointing in the direction of transcription.

The 35 CRISPR‐positive complete genomes and 28 of the 46 CRISPR‐positive high‐quality assemblies were analyzed to identify possible shared genomic contexts. Eight different genomic contexts, named from A to H, were identified. Contexts A to D (Figure 6) were shared by different genomes, while those from E to H were identified in single genomes. The genomic context A (*mdtN*‐*phnP*) has previously been described in *S*. *marcescens* strains isolated as a secondary symbiont of RPW and in other *marcescens* complete genomes available in the NCBI database (Scrascia et al., 2019) becoming the most commonly shared in this study being identified in 55 genomes distributed as follows: 35 *marcescens*, one *grimesii*, one *inhibens*, one *nematodiphila*, six *plymuthica*, six *rubidaea*, and five *Serratia* sp. Contexts B (*puu* genes‐*mnmA*), C (*osmE*‐*soxG*), and D (*ampC*‐*yebZ*) were shared by 11, four, and six genomes, respectively; context B by genomes of species *fonticola* (2), *rubidaea* (7), and *Serratia* sp. (2); C and D only by *rubidaea* genomes. For context D, assignment to *rubidaea* was assumed for the strain JUb9 (see above). The contexts E (*nrdG*‐*bglH*) and F (*sucD*‐*vasK*) were both identified in the single genome of *S*. *oryzae* strain J11‐6; while G (*gntR*‐*cda*) and H (*gutQ*‐*queA*) in genomes of the *Serratia* sp. Ag1 and *S*. *symbiotica* CWBI‐2.3, respectively (Table 3). Distribution of the genomic contexts by subtypes of cas gene sets and/or consensus DR types is reported in Table A2. Genomes of species *rubidaea* were characterized by the presence of multiple CRISPR contexts (A, B, C, D) with the context C associated with the cas gene set of subtype I‐C.

**Table 3:** Genomic contexts

Genomic context	Chromosomal region	Species (#genomes)	Strains
A	*mdtN*‐*phnP*	*marcescens* (35)	E28; S5; S8; B3R3; PWN146; CAV1492; 12TM; 2880STDY5682818; 2880STDY5682863; AH0650_Sm1; AR_0130; CAV1761; EGD‐HP20; EL1; FZSF02; KS10; MC459; 2880STDY5682911; 2880STDY5683032; 2880STDY5682819; 2880STDY5682934; 2880STDY5682957; 2880STDY5682995; 454_SMAR; 420_SMAR; 395_SMAR; 370_SMAR; 1145_SMAR; MSB1_9C‐sc‐2280320; N4‐5; SER00094; SCQ1; SM03; MGH136; at10508;
		*grimesii* (1)	NBRC 13537
		*inhibens* (1)	PRI‐2c
		*nematodiphila* (1)	DH‐S01
		*plymuthica* (6)	AS9; AS12; AS13; NCTC8015; NCTC8900; V4
		Unknown (5)	TEL; SSNIH1; KUDC3025; MYb239; JUb9
		*rubidaea* (6)	FGI94; NCTC10848; FDAARGOS_880; NCTC10036; NCTC12971; FDAARGOS_926
B	*puu* genes‐*mnmA*	*fonticola* (2)	DSM 4576; 5 l
		*rubidaea* (7)	NCTC10848; FDAARGOS_880; NCTC9419; NCTC10036; NCTC12971; FDAARGOS_926; FGI94
		Unknown (2)	JUb9; MYb239
C	*osmE*‐*sox*G	*rubidaea* (4)	NBRC 103169; CFSAN059619; NCTC12971; FDAARGOS_926
D	*ampC*‐*yebZ*	*rubidaea* (5)	FDAARGOS_926; NCTC12971; NCTC10036; NCTC9419; FDAARGOS_880;
		Unknown (1)	JUb9
E	*nrdG*‐*bglH*	*oryzae* (1)	J11‐6
F	*sucD*‐v*asK*		
G	*gntR*‐*cda*	Unknown (1)	Ag1
H	*gutQ*‐*queA*	*symbiotica* (1)	CWBI‐2.3

## 4 DISCUSSION

Bacteria of the genus *Serratia* are ubiquitous and have been isolated from soil, water, plant roots, insects, and the gastrointestinal tract of animals (Cristina et al., 2019; Gupta et al., 2021; Lo et al., 2016). This broad range of environments exposes *Serratia* strains to exogenous genetic elements such as plasmids, phages, and chromosomal fragments of other bacteria. Some of them may represent a life threat (e.g., phages) or a metabolic burden (e.g., plasmids) to which CRISPR–Cas systems represent a unique adaptative immunity defense mechanism. Studying the presence/absence of CRISPR–Cas systems and their features in different genera of families is a relatively new scientific approach to investigation to gain data on the evolution of these systems and their role played during the bacterial lifetime (Butiuc‐Keul et al., 2022). The average percentage of CRISPR distribution among Bacteria is the outcome of processes and/or factors that play different ecological roles within a genus/species. Among these processes/factors are noteworthy the balance between protection provided by CRISPR systems and their possible deleterious effects (e.g., self‐targeting spacers), the role played by exogenous genetic elements (e.g., plasmids, phages, etc.) in bacteria evolution and the horizontal transfer of CRISPR systems.

Data on CRISPR loci in *Serratia* are limited to complete genomes of *S*. *marcescens* strains (Medina‐Aparicio et al., 2018; Scrascia et al., 2019; Srinivasan & Rajamohan, 2019; Vicente et al., 2016). In the present study, along with the species *marcescens*, we extended data on CRISPR loci to 14 additional *Serratia* species. Note, CRISPRs were detected in 24% of the complete genomes and about 14% of the high‐quality assemblies analyzed. The percentage of detection is lower than that reported for Bacteria (about 40%) (Makarova et al., 2020). However, whether the lower percentage of detection in *Serratia* reflects a distinguishing feature of the genus (particularly for the most representative analyzed *marcescens* species where the percentage was 12.6%) or a misrepresentative distribution of the available genomes in databases, remains to be established.

Most of the loci identified in this study were located within the genomic context *mdtN*‐*phnP* previously reported in the species *marcescens* and now further extended to those of *grimesii*, *inhibens*, *nematodiphila*, *plymuthica*, and *rubidaea*. Three new possible contexts were also identified: one (*puu* genes*‐mnmA*) shared by genomes of *rubidaea* and *fonticola*; and two (*osmE*‐*soxG* and *ampC*‐*yebZ*) detected in those of *rubidaea*. The context *osmE*–*soxG* might be closely linked to the cas gene set of subtype I‐C (Table A2). Due to the low number of CRISPR‐positive genomes of *rubidaea* and *fonticola* and genomes positive for the cas gene set I‐C, further analyses are required to confirm this hypothesis.

A previous comprehensive study on the distribution of CRISPR–Cas systems in genomes of the *Enterobacteriaceae* family (now reorganized within the Enterobacterales order) showed the predominant presence of subtype I‐E and the rare coexistence of subtypes I‐E and I‐F1 in the same genome (Medina‐Aparicio et al., 2018). Our data show the prevalence of subtype I‐F1 (39.5%), followed by subtypes I‐E (about 5%), and I‐C (about 5%). Detection of subtype I‐C is the first report in *Serratia*. The prevalence of the subtype I‐F1 in our subset of CRISPR‐positive genomes is consistent with both the new reorganized Enterobacterales order (Adeolu et al., 2016) and data produced by Medina‐Aparicio et al. (2018). Indeed, in the aforementioned study subtype I‐F1 was found prevalent in genera *Yersinia*, *Rahnella*, and *Serratia* which are now part of the new *Yersiniaceae* family. On the other hand, the subtype I‐E remains predominant within the *Enterobacteriaceae* family. Moreover, the finding of two distinct cas‐gene sets (I‐E/I‐F1 or I‐E*/I‐F1) in only three *Serratia* genomes, confirms that the coexistence of these subtypes is not frequent. It is also important to note that the only *Serratia* strain harboring a type III system reported by Medina‐Aparicio et al. (2018) is ATCC 39006. This strain was not included in our study due to recommendations stated by Sandner‐Miranda et al. (2018) which highlighted the need to revise the assignment of the above‐mentioned strain to the *Serratia* genus. In this respect, it is noteworthy that in any complete genomes and high‐quality assemblies considered in our study, the type III system was not detected.

Six different cas‐gene set architectures were identified of which those reported as I‐E unique locus 1 (characterized by a 0.6 kb *cas3*/*cas8e* intergenic sequence), I‐E* (characterized by the *cas6e* translocation between *cas7* and *cas11*) and I‐F1 unique locus 1 (characterized by 0.4 kb *cas3*/*cas8f1* intergenic sequence) are, to the best of our knowledge, the first ever detected in *Serratia*. Similar or identical architectures of I‐E unique locus 1, I‐E*, and I‐F1 unique locus 1 have been reported for other bacteria genera: a similar architecture to I‐E unique locus 1 has been described in *Escherichia coli* (IGLB fragment) where the *cas3*/*cas8e* intergenic sequence was ~0.4 kb (Pul et al., 2010; Westra et al., 2010); an architecture identical to I‐E* has already been detected in *Klebsiella* and *Vibrio* (I‐E variant) strains (McDonald et al., 2019; Shen et al., 2017); a similar architecture to I‐F1 unique locus 1 was reported in *V. cholerae* (I‐FV1), where the *cas3*/*cas8f1* intergenic sequence was ~0.1 kb (McDonald et al., 2019).

This study also supplies data on the presence/number of CRISPRs and their consensus DRs sequences in *Serratia*. Apart from canonical arrays (61.5% of the total disclosed arrays), orphans (29.4%) and aliens (10.2%) arrays were also detected (Table 1; Figure 1). Orphan arrays might represent remnants of previous complete CRISPR–Cas systems (Zhang & Ye, 2017). The presence of alien arrays found only in *rubidaea* complete genomes is, as far as we know, the first report in bacteria CRISPR‐positive genomes. Its detection might be explained as traces of ancient complete CRISPR–Cas systems I‐E/I‐F1 or I‐C/I‐E/I‐F1 coexistent within the same genome (Table 1). Alternatively, the aliens might result from single horizontal gene transfer events. Further analyses could unveil their genetic origin and the entity of their distribution among CRISPR‐positive bacteria genomes. Detection of more alien arrays might unveil that the presence of multiple subtypes in a genome is more frequent than it has been reported so far. Furthermore, consensus DRs specifically associated with the cas gene set I‐E* were also first described (Table 2).

Finally, the phylogenetic tree generated by multiple alignment of the Cas3 sequences showed a potential sub‐lineage (I‐E*) within the I‐E branch and thus might represent and/or anticipate a distinct clonal expansion of an I‐E sub‐population (Figure 4).

Knowledge of CRISPR–*Cas* systems is constantly expanding due to studies on newly available genomic sequences or genomic sequences not yet explored. The CRISPR–Cas systems classification is thus continuously updating also in light of their possible applications. Indeed, the CRISPR–Cas technology has undoubtedly revolutionized systems of genome editing with a wide range of potential industrial and biomedical applications. Other, more recent genome‐editing tools are based on methods that make use of the Cas9 protein (Arroyo‐Olarte et al., 2021). However, expression of foreign proteins with DNA‐binding and editing activity appears toxic for many bacteria. Harness of endogenous CRISPR systems is a recent and promising new line of approach for bacteria genome editing (Klompe et al., 2019; Strecker et al., 2019).

Our study has contributed to expanding knowledge of the variability and distribution of CRISPR systems in the *Serratia* genus. Data here presented might be exploitable for native CRISPR effectors of this genus that includes species (e.g., *marcescens*) relevant in environmental and clinical fields. Moreover, the detection of the same subtype of cas‐gene sets in different *Serratia* species and other genera highlights the open question of the molecular mechanisms yet to be identified that have allowed intra‐ and inter‐species spread.

## AUTHOR CONTRIBUTIONS

**Maria Scrascia:** Conceptualization (equal); investigation (equal); methodology (equal); writing – original draft (equal); writing – review and editing (equal). **Roberta Roberto:** Formal analysis (equal); investigation (equal). **Pietro Daddabbo:** Formal analysis (equal). **Yosra Ahmed:** Data curation (equal). **Francesco Porcelli:** Conceptualization (equal). **Marta Oliva:** Investigation (equal). **Carla Calia:** Investigation (equal). **Angelo Marzella:** Investigation (equal). **Carlo Pazzani:** Methodology (equal); supervision (equal); writing – original draft (equal); writing – review and editing (equal).

## CONFLICT OF INTEREST

None declared.

## ETHICS STATEMENT

None required.

## Supporting information

Table S1: List of *Serratia* genome assemblies.

Table S2: Spacer analyses.

Figure S1: Phylogenetic tree of 16S rRNA gene.

## Data Availability

All data supporting the findings of this study are available within the article (Appendix) and its Supporting Information files (Supporting Information: Table S1: List of *Serratia* genome assemblies; Supporting Information: Table S2: Spacer analyses; Supporting Information: Figure S1: Phylogenetic tree of 16S rRNA gene). Sequences used to generate the 16S tree are available via the reported accession numbers of all analyzed strains; cas gene sequences are available via the CRISPR–Cas^++^ database at https://crisprcas.i2bc.paris-saclay.fr/MainDb/StrainList.

## 1

**Table A1:** cas gene set positive contigs/scaffolds

Subtype of cas gene cluster	Species	Strain	Source	Place of isolation	Year of isolation	Assembly level	Accession/Assembly
I‐C	*rubidaea*	NBRC 103169	N/A	N/A	N/A	Contig	GCA_001598675.1
I‐C	*rubidaea*	CFSAN059619	Throat	Pakistan	1998	Contig	NZ_JACYQC010000002
I‐E	*marcescens*	S8	*Rhynchophorus ferrugineus*	Italy	2013	Contig	MK507744
I‐E	*marcescens*	AH0650_Sm1	clinical	Australia	2014	Contig	GCA_001051865.1
I‐E	*marcescens*	EGD‐HP20	tannery waste	India	2005	Contig	GCA_000465615.2
I‐E unique locus 1	*marcescens*	2880STDY5683025	clinical	United Kingdom	2011	Scaffold	GCA_001538785.1
I‐E unique locus 1	*marcescens*	ML2637	clinical	South Africa	2016	Scaffold	GCA_002118055.1
I‐E*	*Serratia* sp.	DD3^c^	*Daphnia magna*	Germany	2008	Contig	GCA_000496755.2
I‐F1	*marcescens*	2880STDY5682818	blood	United Kingdom	2002	Scaffold	GCA_001539025.1
I‐F1	*marcescens*	2880STDY5682863	blood	United Kingdom	2004	Scaffold	GCA_001539585.1
I‐F1	*marcescens*	MC620	clinical	United States	N/A	Scaffold	GCA_000418815.1
I‐F1	*marcescens*	MC6001	clinical	United States	N/A	Scaffold	GCA_000418835.1
I‐F1	*marcescens*	MC6000	clinical	United States	N/A	Scaffold	GCA_000418855.2
I‐F1	*marcescens*	MC460	clinical	United States	N/A	Scaffold	GCA_000418875.1
I‐F1	*marcescens*	MC459	clinical	United States	N/A	Scaffold	GCA_000418895.1
I‐F1	*marcescens*	MC458	clinical	United States	N/A	Scaffold	GCA_000418915.1
I‐F1	*marcescens*	AB42556419‐isolate1	clinical	United States	N/A	Scaffold	GCA_000418935.1
I‐F1	*marcescens*	2880STDY5682911	clinical	United Kingdom	2006	Scaffold	GCA_001537545.1
I‐F1	*marcescens*	2880STDY5683032	clinical	United Kingdom	2006	Scaffold	GCA_001538705.1
I‐F1	*marcescens*	2880STDY5682819	clinical	United Kingdom	2006	Scaffold	GCA_001537145.1
I‐F1	*marcescens*	2880STDY5682934	clinical	United Kingdom	2007	Scaffold	GCA_001538745.1
I‐F1	*marcescens*	2880STDY5682957	clinical	United Kingdom	2008	Scaffold	GCA_001540825.1
I‐F1	*marcescens*	2880STDY5682995	clinical	United Kingdom	2010	Scaffold	GCA_001537925.1
I‐F1	*marcescens*	684_SMAR	clinical	United States	2012–2013	Scaffold	GCA_001065935.1
I‐F1	*marcescens*	SM03	clinical	India	2012	Scaffold	GCA_001909165.1
I‐F1	*marcescens*	MGH136	clinical	United States	2015	Scaffold	GCA_002153355.1
I‐F1	*marcescens*	at10508	clinical	Australia	2017	Scaffold	GCA_002250685.1
I‐F1	*marcescens*	907_SMAR	clinical	United States	2012–2013	Contig	GCA_001068085.1
I‐F1	*marcescens*	1145_SMAR	clinical	United States	2012–2013	Scaffold	GCA_001060335.1
I‐F1^a^	*fonticola*	5 l	*Alces alces* from permafrost	Russia	2010	Contig	GCA_001908045.1
I‐F1	*Serratia* sp.	HMSC15F11	clinical	N/A	N/A	Scaffold	GCA_001808215.1
I‐F1	*Serratia* sp.	TEL	soil	South Africa	2014	Contig	GCA_001011075.1
I‐F1^b^	*Serratia* sp.	H1w	Phytotelma	Malaysia	N/A	Contig	GCA_000633355.1
I‐F1^b^	*Serratia* sp.	H1n	Phytotelma	Malaysia	N/A	Contig	GCA_000633315.1
I‐F1 unique locus 1	*marcescens*	410_SMAR	clinical	United States	2012–2013	Scaffold	GCA_001063325.1
I‐F1 unique locus 1	*marcescens*	374_SMAR	clinical	United States	2012–2013	Scaffold	GCA_001064725.1
I‐F1 unique locus 1	*marcescens*	454_SMAR	clinical	United States	2012–2013	Scaffold	GCA_001064975.1
I‐F1 unique locus 1	*marcescens*	420_SMAR	clinical	United States	2012–2013	Scaffold	GCA_001063375.1
I‐F1 unique locus 1	*marcescens*	398_SMAR	clinical	United States	2012–2013	Scaffold	GCA_001064855.1
I‐F1 unique locus 1	*marcescens*	395_SMAR	clinical	United States	2012–2013	Scaffold	GCA_001064835.1
I‐F1 unique locus 1	*marcescens*	370_SMAR	clinical	United States	2012–2013	Scaffold	GCA_001064715.1
I‐F1 unique locus 1	*marcescens*	S5	*Rhynchophorus ferrugineus*	Italy	2014	Contig	MK507745
I‐F1 unique locus 1	*grimesii*	NBRC 13537	N/A	N/A	N/A	Contig	GCA_001590905.1
I‐E; I‐F1	*oryzae*	J11‐6	rice	China	2015	Scaffold	GCA_001976145.1
I‐E*; I‐F1	*Serratia* sp.	Ag1^d^	*Anopheles gambiae*	France	2014	Contig	GCA_000743355.1
I‐E*; I‐F1	*Serratia* sp.	Ag2^e^	*Anopheles gambiae*	United States	2014	Contig	GCA_000743365.1

Abbreviation: N/A, not applicable.

^a^ Stop codon detected in the gene *cas8f*.

^b^ Truncated sequence: flanking regions of the identified set of cas genes were not completely available.

^c^ Two arrays (26 DRs and 45 DRs) were detected.

^d^ Four arrays (5 DRs, 16 DRs, 20 DRs, and 27 DRs) were detected.

^e^ Four arrays (3 DRs, 5 DRs, 16 DRs, and 27 DRs) were detected.

**Table A2:** Distribution of genomic contexts

Subtype of cas gene cluster	CRISPRs		Species	Strain	Source	Place of isolation	Year of isolation	Assembly level	Accession/Assembly	Genomic contexts
	ConsensusDR type	#Arrays (#repeats)								
I‐C	I‐C	1 (15)	*rubidaea*	FDAARGOS_926^a^	N/A	N/A	N/A	Complete genome	NZ_CP065640.1	C
	I‐E	1 (8)								A
	I‐F	1 (6)								B
	I‐F	1 (3)								D
I‐C	I‐C	1 (15)	*rubidaea*	NCTC12971^a^	N/A	N/A	N/A	Complete genome	LR590463.1	C
	I‐E	1 (8)								A
	I‐F	1 (6)								B
	I‐F	1 (3)								D
I‐C	N/A	N/A	*rubidaea*	CFSAN059619	Throat	Pakistan	1998	Contig	NZ_JACYQC010000002	C
I‐C	N/A	N/A	*rubidaea*	NBRC 103169	N/A	N/A	N/A	Contig	BCZJ01000003	C
I‐E	I‐E	2 (44, 31)	*plymuthica*	NCTC8900	N/A	N/A	N/A	Complete genome	LR134151.1	A
I‐E	I‐E	3 (11, 30, 31)	*marcescens*	S8	*Rhynchophorus ferrugineus*	Italy	2013	Contig	MK507744	A
I‐E	N/A	N/A	*marcescens*	AH0650_Sm1	Clinical	Australia	2014	Contig	LFJS01000001.1	A
I‐E	N/A	N/A	*marcescens*	EGD‐HP20	Tannery waste	India	2005	Contig	AVSR01000005.1	A
I‐E unique locus 1	I‐E	4 (7, 9, 28, 45)	*marcescens*	E28	Hospital Ensuite	Australia	2012	Complete genome	CP042512.1	A
I‐E unique locus 1	I‐E	3 (8, 11, 23)	*marcescens*	SER00094	Clinical	United States	2017	Complete genome	CP050447.1	A
I‐E unique locus 1	I‐E	3 (12, 40, 70)	*marcescens*	MSB1_9C‐sc‐2280320	N/A	N/A	N/A	Complete genome	LR890657.1	A
I‐E unique locus 1	I‐E	2 (36, 48)	*plymuthica*	NCTC8015	Canal water	N/A	N/A	Complete genome	LR134478.1	A
I‐F1	I‐F	2 (7, 47)	*marcescens*	PWN146	*Bursaphelenchus xylophilus*	Portugal	2010	Complete genome	LT575490.1	A
I‐F1	I‐F	2 (26, 28)	*marcescens*	12TM	Pharyngeal secretions	Romania	2014	Complete genome	CM008894.1	A
I‐F1	I‐F	2 (9, 18)	*marcescens*	N4‐5	Soil	United States	1995	Complete genome	CP031316.1	A
I‐F1	I‐F	2 (4, 51)	*marcescens*	S5	*Rhynchophorus ferrugineus*	Italy	2014	Contig	MK507745	A
I‐F1	N/A	N/A	*marcescens*	2880STDY5682818	Blood	United Kingdom	2002	Scaffold	FCGU01000002.1	A
I‐F1	N/A	N/A	*marcescens*	2880STDY5682863	Blood	United Kingdom	2004	Scaffold	FCHP01000003.1	A
I‐F1	N/A	N/A	*marcescens*	MC459	Clinical	United States	N/A	Scaffold	ATOK01000005.1	A
I‐F1	N/A	N/A	*marcescens*	2880STDY5682911	Clinical	United Kingdom	2006	Scaffold	FCFC01000002.1	A
I‐F1	N/A	N/A	*marcescens*	2880STDY5683032	Clinical	United Kingdom	2006	Scaffold	FCFQ01000002.1	A
I‐F1	N/A	N/A	*marcescens*	2880STDY5682819	Clinical	United Kingdom	2006	Scaffold	FCGD01000002.1	A
I‐F1	N/A	N/A	*marcescens*	2880STDY5682934	Clinical	United Kingdom	2007	Scaffold	FCJR01000002.1	A
I‐F1	N/A	N/A	*marcescens*	2880STDY5682957	Clinical	United Kingdom	2008	Scaffold	FCKI01000003.1	A
I‐F1	N/A	N/A	*marcescens*	2880STDY5682995	Clinical	United Kingdom	2010	Scaffold	FCLS01000002.1	A
I‐F1	N/A	N/A	*marcescens*	454_SMAR	Clinical	United States	2012–2013	Scaffold	JVGM01000005.1	A
I‐F1	N/A	N/A	*marcescens*	420_SMAR	Clinical	United States	2012–2013	Scaffold	JVHU01000010.1	A
I‐F1	N/A	N/A	*marcescens*	395_SMAR	Clinical	United States	2012–2013	Scaffold	JVIU01000009.1	A
I‐F1	N/A	N/A	*marcescens*	370_SMAR	Clinical	United States	2012–2013	Scaffold	JVJT01000036.1	A
I‐F1	N/A	N/A	*marcescens*	SM03	Clinical	India	2012	Scaffold	LZOB01000021.1	A
I‐F1	N/A	N/A	*marcescens*	MGH136	Clinical	United States	2015	Scaffold	NGUE01000001.1	A
I‐F1	N/A	N/A	*marcescens*	at10508	Clinical	Australia	2017	Scaffold	NPIX01000022.1	A
I‐F1	N/A	N/A	*marcescens*	1145_SMAR	Clinical	United States	2012–2013	Scaffold	JWBL01000004.1	A
I‐F1	I‐F	2 (8, 18)	*fonticola*	5 l	*Alces alces* from permafrost	Russia	2010	Contig	MQRH01000015.1	B
I‐F1	I‐F	4 (12, 16, 23, 72)	*fonticola*	DSM 4576	Water	N/A	1979	Complete genome	NZ_CP011254.1	B
I‐F1	I‐F	2 (16, 25)	*inhibens*	PRI‐2c	Maize rhizosphere soil	The Netherlands	2004	Complete genome	NZ_CP015613.1	A
I‐F1	I‐F	6 (2, 8, 8, 15)	*rubidaea*	FDAARGOS_880	N/A	N/A	N/A	Complete genome	CP065717.1	A
	I‐F	1 (15)								B
	I‐F	1 (4)								D
I‐F1	N/A	N/A	*Serratia* sp.	TEL	Soil	South Africa	2014	Contig	LDEG01000006.1	A
I‐F1 unique locus 1	I‐F	3 (6, 11, 30)	*marcescens*	FZSF02	Soil	China	2014	Complete genome	CP053286	A
I‐F1 unique locus 1	I‐F	3 (4, 7, 8)	*rubidaea*	NCTC10036	Finger	N/A	N/A	Complete genome	LR134493.1	A
	I‐F	1 (9)								B
	I‐E	1 (4)								D
I‐F1 unique locus 1	I‐F	1 (11)	*Serratia* sp.	JUb9	Compost	France	2019	Complete genome	CP060416.1	B
	I‐F	3 (3, 8, 8)								A
	I‐F	1 (3)								D
I‐F1 unique locus 1	I‐F	2 (16, 17)	*rubidaea*	FGI94	*Atta colombica*	Panama	2009	Complete genome	NC_020064.1/CP003942.1	A
	I‐E	1 (10)								A
	I‐F	1 (7)								B
I‐F1 unique locus 1	N/A	N/A	*grimesii*	NBRC 13537	N/A	N/A	N/A	Contig	BCTT01000008.1	A
I‐E	N/A	N/A	*oryzae*	J11‐6	Rice	China	2015	Scaffold	MOXD01000003.1	F
I‐F1								Scaffold	MOXD01000008.1	E
I‐E*	I‐E*	2 (5, 20)	*Serratia* sp.	Ag1	*Anopheles gambiae*	France	2014	Contigs	JQEI01000052.1; JQEI01000046.1	N/A
I‐F1	I‐F	2 (16, 27)						Contig	JQEI01000002.1	G
N/A	I‐C	1 (10)	*symbiotica*	CWBI‐2.3	*Aphis fabae*	Belgium	2009	Complete genome	GCA_000821185.1	H
N/A	I‐E	1 (27)	*marcescens*	KS10^b^	Marine	United States	2006	Complete genome	CP027798.1	A
N/A	I‐E	1 (27)	*marcescens*	EL1^b^	Marine	United States	2002	Complete genome	CP027796.1	A
N/A	I‐E	1(39)	*marcescens*	CAV1492	Clinical	United States	2011–2012	Complete genome	NZ_CP011642.1	A
N/A	I‐E	2 (4, 34)	*marcescens*	CAV1761	Peri‐rectal	Virginia	2014	Complete genome	CP029449.1	A
N/A	I‐E	1 (3)	*Serratia* sp.	KUDC3025	Rhizospheric soil	South Korea	2017	Complete genome	CP041764.1	A
N/A	I‐F	1 (22)	*marcescens*	SCQ1	Blood from silkworm	China	2009	Complete genome	CP063354.1	A
N/A	I‐F	1 (4)	*marcescens*	AR_0130	N/A	N/A	N/A	Complete genome	CP028947.1	A
N/A	I‐F	1 (4)	*marcescens*	B3R3	*Zea mays*	China	2011	Complete genome	NZ_CP013046.2	A
N/A	I‐F	1 (4)	*nematodiphila*	DH‐S01	N/A	N/A	N/A	Complete genome	CP038662.1	A
N/A	I‐F	1 (7)	*plymuthica*	AS9^c^	Plant	Sweden	N/A	Complete genome	NC_015567.1	A
N/A	I‐F	1 (7)	*plymuthica*	AS12^c^	Plant	Sweden	1998	Complete genome	NC_015566.1	A
N/A	I‐F	1 (7)	*plymuthica*	AS13^c^	Plant	Sweden	N/A	Complete genome	NC_017573.1	A
N/A	I‐F	1 (3)	*plymuthica*	V4	Milk processing plant	Portugal	2006	Complete genome	CP007439.1	A
N/A	I‐F	1 (2)	*Serratia* sp.	MYb239	Compost	Germany	N/A	Complete genome	CP023268.1	A
	I‐F	1 (3)								B
N/A	I‐F	1 (4)	*Serratia* sp.	SSNIH1	N/A	United States	2015	Complete genome	CP026383.1	A
N/A	I‐F	1 (5)	*rubidaea*	NCTC9419	N/A	N/A	N/A	Complete genome	LR134155.1	B
	I‐F	1 (7)								D
N/A	I‐F	1 (3)	*rubidaea*	NCTC10848	N/A	N/A	N/A	Complete genome	LS483492.1	A
	I‐E	1 (4)								A
	I‐F	1 (7)								B

Abbreviation: N/A: not applicable.

^a,b,c^ Possible multiple records of the same genome. Spacers’ sequences were identical.
