# Supplementary figures and images for: Bioinformatic survey of CRISPR loci across 15 Serratia species
Source: Microbiologyopen. 2023 Mar 2;12(2):e1339. doi: 10.1002/mbo3.1339 (PMC9981886; doi:10.1002/mbo3.1339)

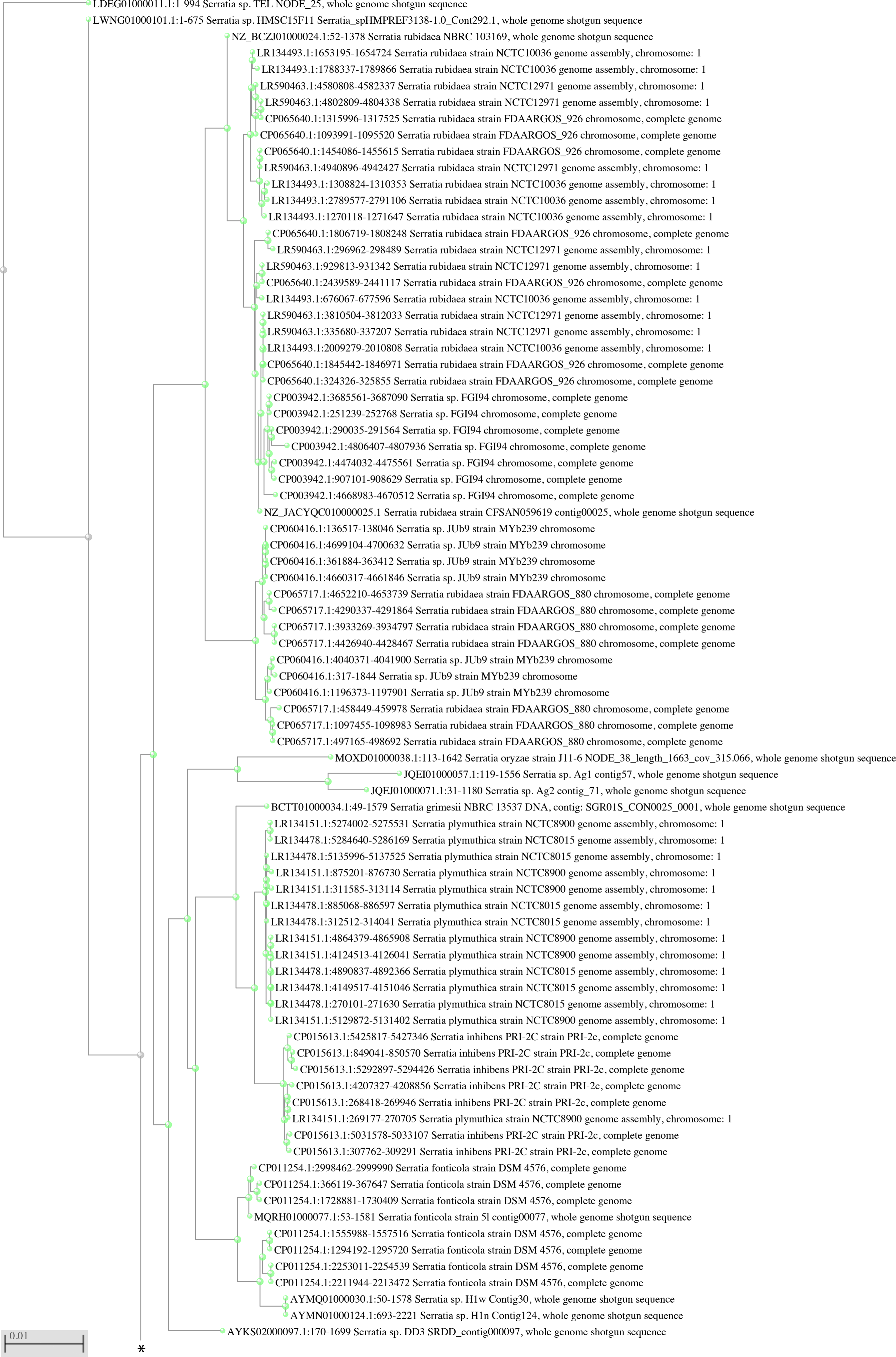

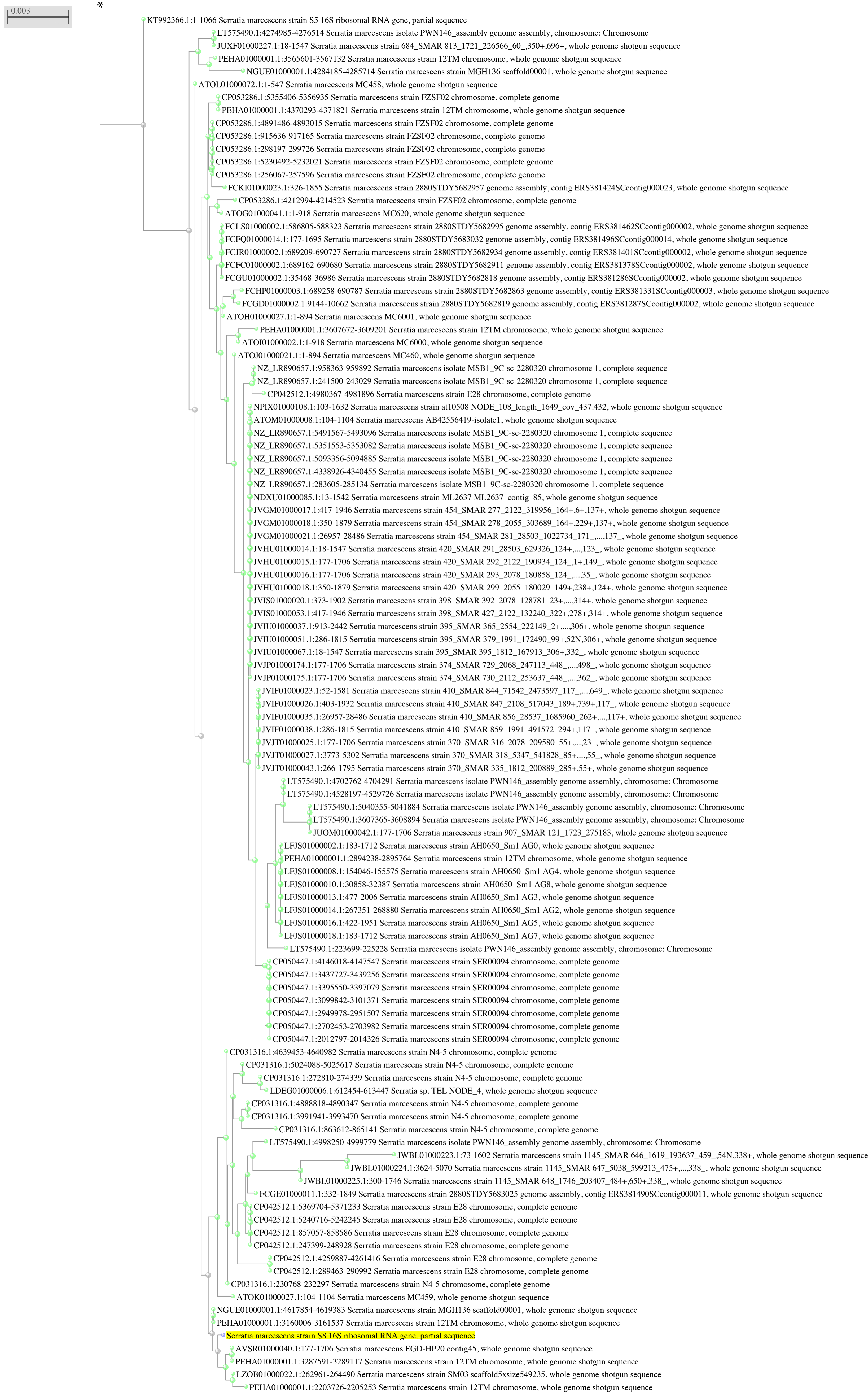

Supplement: Supplementary file 3 — Figure S1: Phylogenetic tree of 16S rRNA gene. [file MBO3-12-e1339-s003.pdf]
